# Cross-sectional study characterizing the porcine faecal microbiome in commercial farms

**DOI:** 10.1186/s40813-025-00480-3

**Published:** 2026-01-22

**Authors:** Mario Andre S. Ornelas, Juan M. Ortiz Sanjuán, Finola C. Leonard, Carla Correia-Gomes, Jordi Estellé, Lorcan O’Neill, Edgar Garcia Manzanilla

**Affiliations:** 1 Pig and Poultry Research and Knowledge Transfer Department, Teagasc Animal and Grassland Research and Innovation Centre, Moorepark, Fermoy, Cork, P61 C996 Ireland; 2 School of Veterinary Medicine, University College Dublin, Belfield, Dublin, Ireland; 3 Animal Health Ireland, Carrick on Shannon, N41 WN27 Ireland; 4 GABI, INRA, Université Paris-Saclay, Jouy-en-Josas, 78350 AgroParisTech France

**Keywords:** Feed, Microbiota, Pig herds, Production and health factors, Resistome, *Salmonella* spp

## Abstract

**Background:**

The gut microbiome is regarded as an important source of information to better understand and improve health and growth in animals. However, there is a knowledge gap concerning the effects of management and health in commercial farms on the porcine faecal microbiome. This study aimed to identify the main factors associated with differences in microbiota alpha and beta diversity, resistome and functional profiles between and within farms and to study the associations between microbiome and farm characteristics. The faecal microbiomes at four production stages were examined in 18 farms, using shotgun sequencing.

**Results:**

Microbiota richness increased with age while evenness and Simpson diversity decreased. Production stage was associated with microbiota composition, resistome and functional profiles, and most differences in the microbiome were observed between weaned pigs and older animals. Microbiota richness, diversity and evenness were higher in farms that purchased feed and in those with lower salmonella prevalence. Microbiota composition was associated with use of zinc oxide and medicated feed in young pigs and with health, biosecurity and feed form in older pigs. Feed form was associated with resistome, functional profiles and especially with core microbiota, as dry fed pigs showed higher abundances of *Streptococcus alactolyticus*.

**Conclusions:**

Consistent patterns of alpha and beta diversity were observed across farms with contrasting characteristics, suggesting that the faecal microbiota in commercial pig farms is primarily driven by diet and age, rather than health. Associations between microbiome, salmonella prevalence and diet suggested that diet induced changes in the microbiome may be a determining factor in gut colonization and infection by pathogens.

**Supplementary Information:**

The online version contains supplementary material available at 10.1186/s40813-025-00480-3.

## Background

The study of the gut microbiota has gained a great deal of attention as a means to understand and improve the health of humans and animals. A strong motivation for this is the pivotal role that gut microbiome plays in immunity [1, 2] and nutrient metabolism [3, 4]. Thanks to recent advances in high throughput sequencing methods and bioinformatics, a vast network of associations between gut microbiome and health have been described in different animals [2, 5, 6]. As an important livestock species and a model for humans, pigs have been extensively studied in terms of their gut microbiome [7–9]. Some areas of pig production which have been substantially explored using high-throughput sequencing include post-weaning gut dysbiosis [10, 11], antimicrobial resistance [12, 13] and nutrient metabolism [3, 14].

Microbial colonization of the porcine gut after birth is mainly shaped by the microbial composition of colostrum and milk, along with other environmental factors such as the sow’s vaginal, faecal and skin microbiota [15]. Early weaning in pig farms directly impacts the gut microbiota, and may cause reduced growth, diarrhoea and death [16, 17]. While the mechanisms behind post-weaning diarrhoea are not yet fully understood, sequencing data have played an important role in advancing knowledge in this area [10]. Following rapid changes at weaning, the microbiota evolves towards stability, influenced by host and environmental factors. Age and diet are among the main factors explaining variability in microbiome studies, and it is generally accepted that microbiota alpha-diversity increases with age [18, 19]. Likewise, several meta-analyses have suggested the existence of a core microbiota across studies [8, 18, 19]. Other factors that have been linked with the composition of microbiota in pig farms include infectious disease [20, 21], antimicrobial use [12, 22], social stress [23] and hygiene [24].

The vast majority of microbiome studies have been carried out in research facilities with a high health status and under controlled conditions [8, 19]. While such studies achieve a good control of background variability and are necessary to advance knowledge, it is also relevant to consider the effects of management and health status of commercial farms on the microbiome. This study aimed to identify the main factors explaining differences in microbiota alpha and beta diversity between and within farms and to study the associations between microbiota and farm characteristics. For this, eighteen commercial farms were visited, and faecal samples were collected at four selected production stages. The porcine oral fluid biomarker profile in these farms is described in Ornelas, et al. [25].

## Methods

### Farm selection

Eighteen farrow-to-finish Irish pig farms were selected to take part in the study, as described in detail in Ornelas, et al. [25] and farm characteristics are described in Table 1. Performance data and biosecurity scores (Biocheck.UGent™ [26]) were obtained from the Teagasc Profit Monitor database [27] and the Animal Health Ireland Pig HealthCheck database [28], respectively. Further data were collected during farm visits. Data for each farm included number of pigs produced per sow per year, mortality rate of piglets, weaners and finishers, weaner + finisher mortality (cumulative mortality in the weaner and finisher stages), post-weaning daily gain, age at sale, porcine reproductive and respiratory syndrome (PRRS) status (positive or negative) and post-weaning use of zinc oxide and medicated feed (ZnOAb; yes or no). Data about feed form (dry or liquid) and origin (purchased or home milled), internal and external biosecurity scores were also used. Farms were further characterized at each stage for prevalence of *Salmonella* spp. and for antimicrobial resistance (AMR) using *Escherichia coli* (*E. coli)* as an indicator organism. AMR is used hereafter to indicate the level of fluoroquinolone-resistant, and ESBL and AmpC cephalosporinase-producing *E. coli* detected in the present study.

**Table 1:** Farm characteristics for the 18 herds in the study group

	Mean ± SE	Median (min-max)
Pigs/sow/year	27.9 ± 0.6	27.9 (23–32.3)
Piglet mortality (%)	11.5 ± 0.7	11.3 (5.9–17.2)
Weaner and finisher mortality (%)	5.7 ± 0.6	5.2 (3.3–14.0)
Post-weaning daily gain (grams)	741 ± 17	730 (632–865)
Age at sale (days)	176 ± 3	178 (148–203)
AMR prevalence (%)^1^	45 ± 8	31 (0–100)
Salmonella prevalence (%)^1^	35 ± 9	25 (0–100)
Herds positive to PRRS (%)	60 ± 10	100 (0–100)
External biosecurity score	82 ± 2	82 (65–93)
Internal biosecurity score	63 ± 3	62 (28–83)
Overall biosecurity score	73 ± 2	73 (47–87)

AMR: antimicrobial resistance; PRRS: porcine reproductive and respiratory syndrome; SE: standard error; ZnO: zinc oxide. ^1^AMR prevalence was calculated as the proportion of plates on which resistant isolates were recovered (number of positive plates/8), and prevalence of *Salmonella* spp. was calculated as the proportion of stages positive for *Salmonella* spp. (number of stages positive/4)

### Sampling stages

Each farm was visited once to collect samples from pigs at four stages: one week after weaning (W1), one week prior to transfer to the finishing stage (W2), one week after transfer to the finishing stage (F1) and one week prior to slaughter (F2). While the former three stages capture the effects of weaning and relocation on the microbiota, stage F2 was chosen for being close to slaughter. In Irish pig farms, weaning usually takes place between 28 and 32 days of age and transfer to the finisher facility at around 12 weeks of age. Animals are slaughtered between 22 and 25 weeks of age, with live weights ranging between 110 and 115 kg.

### Environmental sample collection

Collection, processing and analysis of environmental samples for detection of *Salmonella* spp. and AMR was carried out according to the methods described in Ornelas, et al. [25]. Briefly, a pair of cover-socks (EnviroBootie™, Hardy Diagnostics^®^, California, USA) was used to collect samples at each stage by walking in the pens, and disposable boot covers were worn over farm footwear beforehand to minimize contamination. Each pair of socks was transferred to individual sterile plastic bags and transported until refrigeration. Upon arrival at the laboratory, 250 ml of buffered peptone water (Oxoid, Basingstoke, UK) was transferred into each bag, which was followed by hand massaging and incubation at 37 °C for 18 ± 2 h. The incubated pre-enriched cultures were later aliquoted into sterile 10 ml tube containers for culturing in selective media.

### Detection of fluoroquinolone-resistant and ESBL-/AmpC-producing *E coli* and *Salmonella* spp

For the detection of AMR and *Salmonella* spp. isolates, protocols “Isolation of ESBL-, AmpC- and carbapenemase-producing *E coli* from caecal samples” (EURL-AR, 2019) [29] and ISO 6579-1:2017(E) [30] were followed, respectively. All incubation steps were carried out at 37 °C for 18 ± 2 h. The detection of *E. coli* involved sub-culturing the pre-enriched samples onto MacConkey agar (Neogen, Lansing, USA) supplemented with cefotaxime (1 mg/L) (Santa Cruz technologies, Santa Cruz, USA) and ciprofloxacin (1 mg/L) (Santa Cruz technologies, Santa Cruz, USA). Following incubation, presumptive ESBL-/AmpC- producing *E. coli* colonies were sub-cultured onto Tryptone bile X-glucuronide (TBX) agar (Neogen, Lansing, USA) supplemented with cefotaxime (1 mg/L) and/or ciprofloxacin (1 mg/L), respectively, and incubated. Presence or absence of antimicrobial resistant isolates was recorded for each sample from the four stages. A farm could therefore have AMR detected on up to 8 plates, if typical growth was observed on media supplemented with cefotaxime and ciprofloxacin at the four stages. For each farm, the prevalence of AMR was reported as the proportion of plates on which resistant isolates were recovered.

For the detection of *Salmonella* spp., 100 µl of each pre-enriched sample were inoculated in Modified Semi-solid Rappaport-Vassiliadis (MSRV) agar (Neogen, Lansing, USA). If typical growth was present after incubation, it was sub-cultured onto Xylose Lysine Deoxycholate (XLD) (Neogen, Lansing, USA) and Brilliant Green agar (Neogen, Lansing, USA) and incubated. Otherwise, plates were further incubated for 18 h. If suspected colonies of *Salmonella* spp. were observed, at least one colony was sub-cultured onto Brilliance™ Salmonella (Oxoid, Basingstoke, UK) and nutrient agar (Oxoid, Basingstoke, UK) and incubated. When typical growth was present in the former, serological confirmation by slide agglutination testing was carried out using a small amount of culture from the corresponding nutrient agar plate. The prevalence of *Salmonella* spp. for each farm was reported as the proportion of stages positive for *Salmonella* spp.

### Faecal sample collection, DNA extraction, library preparation and sequencing

Five freshly voided faecal samples were randomly collected from different pens at each stage and pooled into a sterile container by transferring 2.5 g of each sample. After homogenization using a sterile steel spatula, the pooled sample was transferred into 1.5 ml microcentrifuge tubes and snap frozen using dry ice. Upon arrival at the laboratory, samples were stored at -80° C until further analysis.

DNA extraction from faecal samples was carried out using the QIAamp PowerFecal Pro DNA Kit (Qiagen, Crawley, West Sussex, UK) in accordance with the manufacturer’s guidelines, using 200 ± 50 mg of faecal material per sample. The DNA concentration was measured using a Qubit 3 fluorimeter (Invitrogen). Paired-end libraries were prepared from the extracted DNA using the Illumina DNA Prep Library Preparation Kit (Illumina Inc., San Diego, CA). The size of each library was examined on an Agilent Technology 21,000 Bioanalyzer with a High Sensitivity DNA chip and sequencing was performed on the Illumina NovaSeq 6000 platform with an S1 flow cell (2 × 150 bp), following the manufacturer’s protocol.

### Bioinformatics analysis

Quality control of raw sequencing reads was performed using fastQC v0.11.8 [31] and multiQC v1.9 [32], and subsequently processed using Trim Galore v0.6.1 [33], thereby trimming reads based on an average quality score threshold of 20, ensuring a minimum read length of 75 base pairs, with an adapter sequence overlap stringency of 5 base pairs. The validated paired-end trimmed reads were then mapped to host and human reference genomes using Bowtie2 v2.4.4 [34], retaining only the unmapped reads for further analysis of the microbiome. Reference genomes were obtained from the Bowtie2 website. The script clumpify.sh from bbmap v38.22 [35] was used to group and sort reads in order to maximise file compression. Taxonomic classification was conducted using MetaPhlAn v4.0.6 [36], using the database mpa_vOct22_CHOCOPhlAnSGB_202212. Number of reads from each species was estimated considering its coverage and genome length (taken from reference genomes). This was performed adding the argument “-t rel_ab_w_read_stats” when running MetaPhlAn. MetaPhlAn SGB profiles were converted to GTDB-taxonomy-based profiles using a customized version of MetaPhlAn utility script “sgb_to_gtdb_profile.py” to retrieve the estimated number of reads from each profile.

Microbiome functional analysis was carried out according to the methods described in Ortiz Sanjuán, et al. [37]. Briefly, HUMAnN v3.0 [38] was used to assign functional profiles, with subsequent regrouping of gene families into MetaCyc metabolic reactions and broader categories using Microeco R package [39]. MetaCyc is a database of metabolic pathways [40]. The abundance of these pathways was determined from UniRef90 gene families. For antimicrobial resistance gene (ARG) annotation, sequencing reads were mapped against the ResFinder database using *k*-mer alignment software KMA for read alignment [41]. The number of reads mapped to each ARG were used in subsequent analyses. For functional and resistome analyses, data were normalized to counts per million (CPM) prior to analyses based on the total number of reads per sample.

### Statistical analysis

All data were processed and analysed using R version 4.4.1 [42], including R packages vegan version 2.6.4 [43], phyloseq version 1.38.0 [44] and ggplot2 version 3.5.1 [45]. For determination of significance, alpha level was set at 0.05. Microbiota composition, alpha diversity and beta diversity were analysed at the species level. Alpha diversity, richness, Shannon diversity, Simpson diversity (1-D) and Pielou evenness were computed and compared between stages with Kruskal-Wallis tests, followed by Dunn’s tests for post-hoc analysis. Bonferroni’s method was used to adjust P values. Analyses were conducted on the read counts of each species per sample, or on the respective Bray-Curtis distance matrix. Prior to analysis, read counts were normalized by calculating relative abundance due to different sequencing depths between samples. Differences in microbiota composition according to stage and farm characteristics were assessed using permutational multivariate analysis of variance (PERMANOVA), and the respective pairwise comparisons, which test for differences amongst the location of the group centroids in the multivariate space. This was done using R functions adonis2 and pairwise.adonis, respectively [46]. Microbiota was modelled by farm characteristic at each stage separately, due to differences between stages in microbiota composition. Principal coordinate analysis (PCoA) was used for graphical representation of groups of samples based on Bray-Curtis distances and Hellinger transformed Bray-Curtis distances. Hellinger transformation was performed in order to avoid overrepresentation of the most abundant species and to assess the contribution of low-abundant species in the microbiota. Clusters of samples were further investigated by Ward-clustering analysis of Bray-Curtis distances. Species composition of each sample was described in terms of relative abundance or read counts and graphically represented through stacked bar plots. Analyses of microbiome functional profiles and resistome followed the same methods as for microbiota. The abundance of reads assigned to each functional pathway and ARG was normalized to counts per million, based on the total number of reads per sample. The envfit function from package vegan was used to analyse factors influencing ordination results. Linear discriminant analysis effect size (LEfSe) was computed to investigate differential microbiota composition between groups, using stage as class and a LDA threshold of 3.5. General linear regression models were used to model alpha diversity at each stage according to farm characteristics. Following univariable analysis of alpha-diversity indicators by farm characteristics for each stage (Additional file 2), variables were assessed for collinearity and selected for multivariable modelling.

## Results

### Microbiota alpha diversity according to stage

**Fig. 1:**
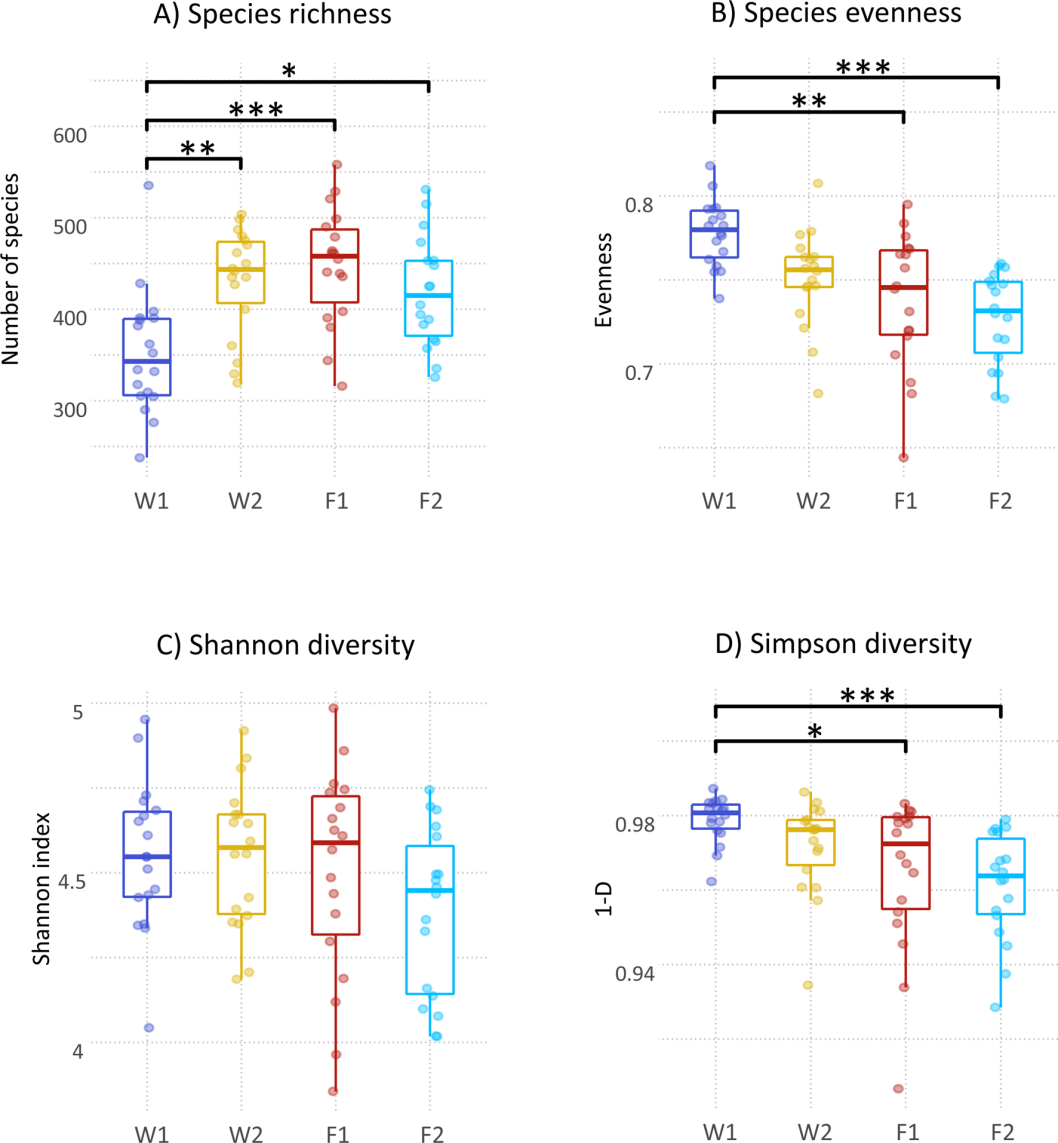
Microbiota species alpha-diversity according to stage reported as: **A**) species richness; **B**) species evenness; **C**) Shannon diversity index and **D**) Simpson diversity index. W1: one week after weaning; W2: one week prior to transfer to the finisher stage; F1: one week after transfer to the finisher stage; F2: one week prior to slaughter; *: *P* < 0.05; **: *P* < 0.01; ***: *P* < 0.001; ****: *P* < 0.0001

**Fig. 2:**
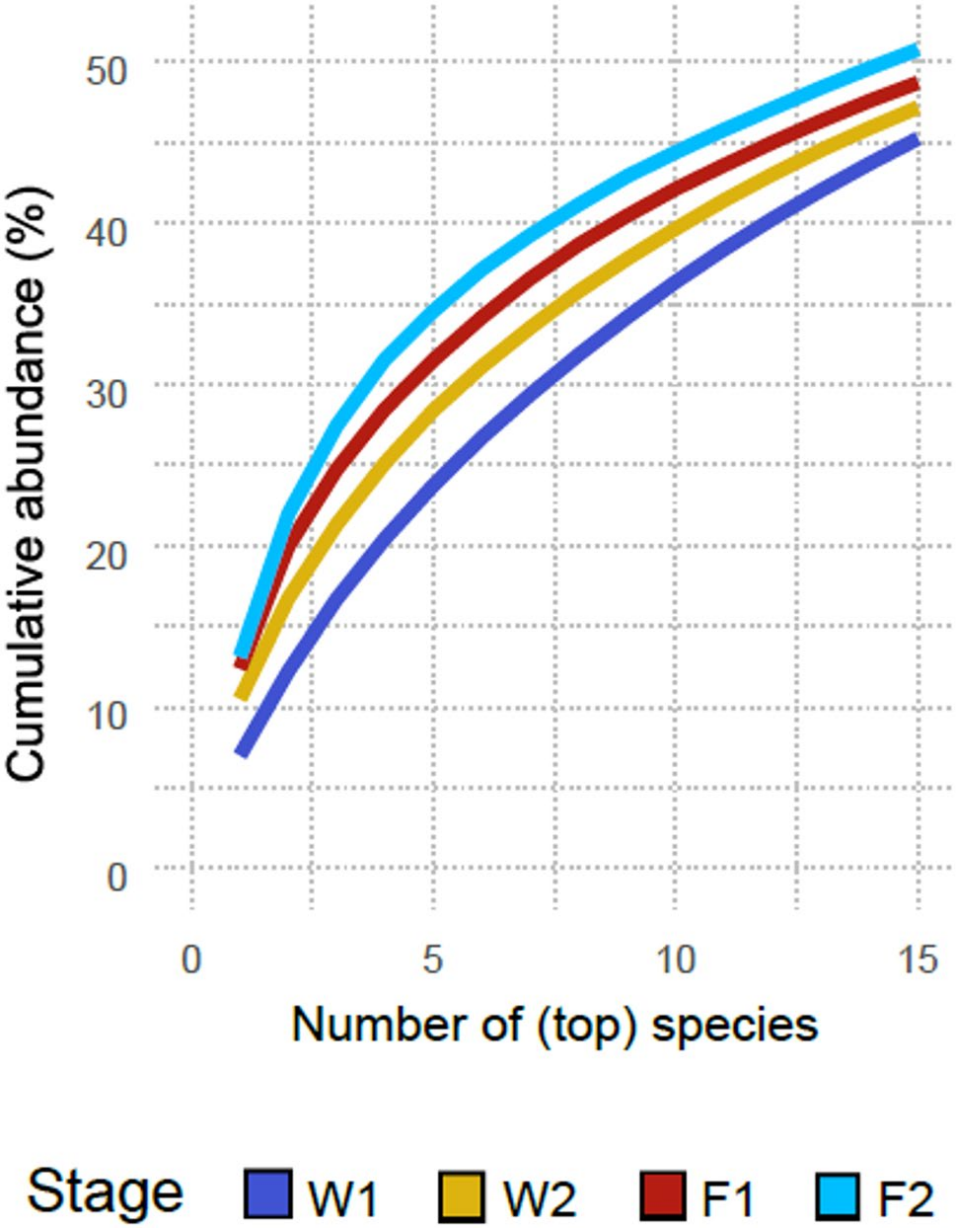
Microbiota cumulative abundance accounted for by the most predominant species at each stage. W1: one week after weaning; W2: one week prior to transfer to the finisher stage; F1: one week after transfer to the finisher stage; F2: one week prior to slaughter

Samples had an average sequencing depth of 29.2 million sequencing reads (range: 13.2 million − 40.5 million). On average, 53% of reads per sample were unclassified and 45% were assigned to species level. Considering all farms, species richness was lowest at the earliest sampling stage (W1), staying relatively constant across the other stages, and F1 had the highest median number of observed species (Fig. 1). Although Shannon diversity did not differ between stages, Simpson diversity (1-D) was highest at W1 and decreased throughout the following stages, denoting a negative association between age and species diversity. Species evenness followed a similar pattern, peaking at W1 and decreasing successively thereafter. The proportion of the microbiota accounted for by the most predominant species increased with age (Fig. 2). While the five most abundant species at W1 represent, on average, 23.8% of the microbiota (SD = 4.8%), the same number of predominant species at F2 accounted for 34.6% of the microbiota (SD = 6.5%). For W2 and F1, the five most predominant species accounted for 28.4% (SD = 5.8%) and 31.5% (SD = 8.3%) of the microbiota, respectively.

### Microbiota composition according to stage

**Fig. 3:**
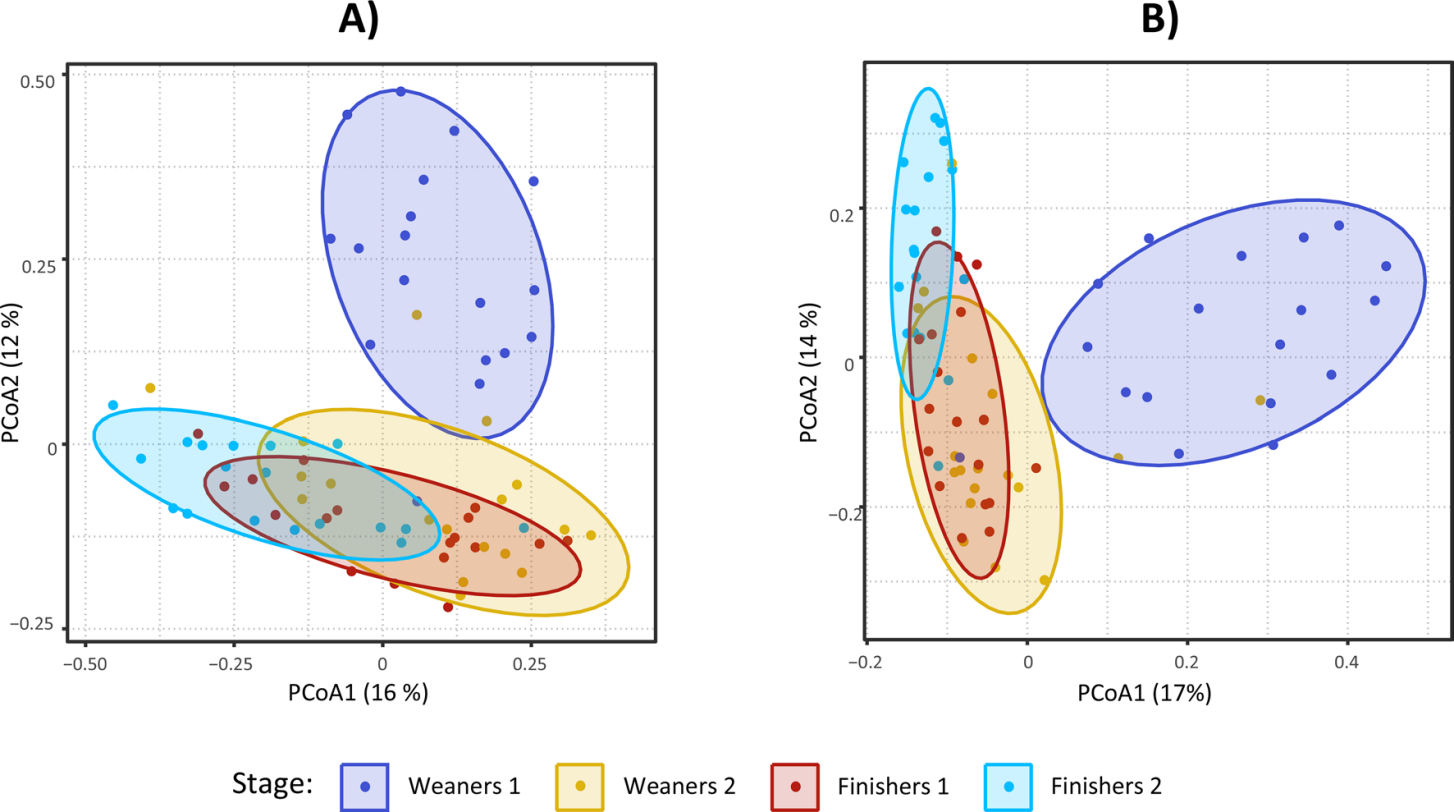
PCoA representation of microbiota composition according to stage based on Bray-Curtis distances of normalized read counts between samples (**A**) and Bray-Curtis distances of Hellinger transformed normalized read counts between samples (**B**). Weaners 1: one week after weaning; Weaners 2: one week prior to transfer to the finisher stage; Finishers 1: one week after transfer to the finisher stage; Finishers 2: one week prior to slaughter

**Fig. 4:**
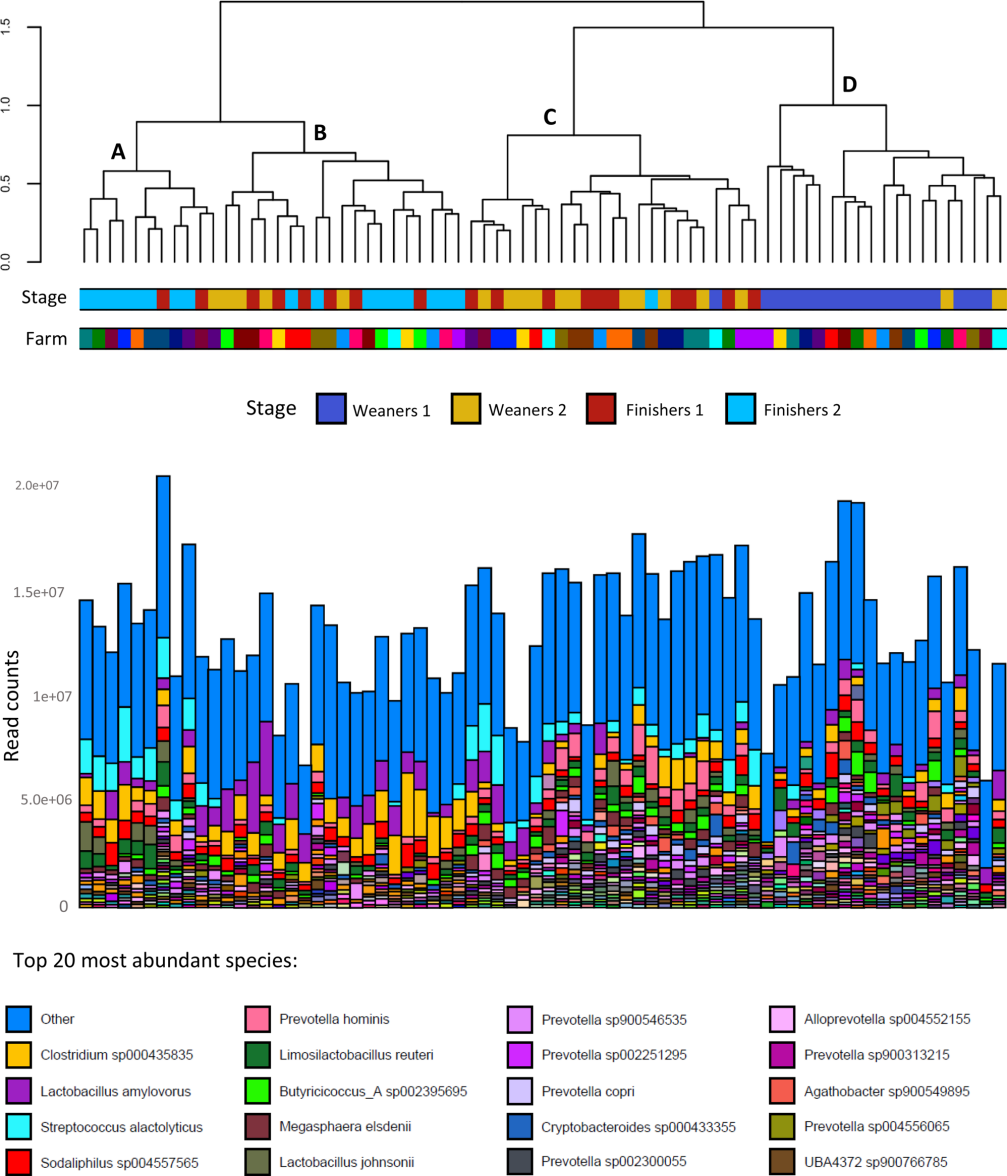
Species abundance of all samples ordered according to Ward-clustering of Br 44247_2025_232ay-Curtis distances of normalized read counts between samples. Weaners 1: one week after weaning; Weaners 2: one week prior to transfer to the finisher stage; Finishers 1: one week after transfer to the finisher stage; Finishers 2: one week prior to slaughter. Note: “Other” includes all species with less than 125,000 read counts

**Fig. 5:**
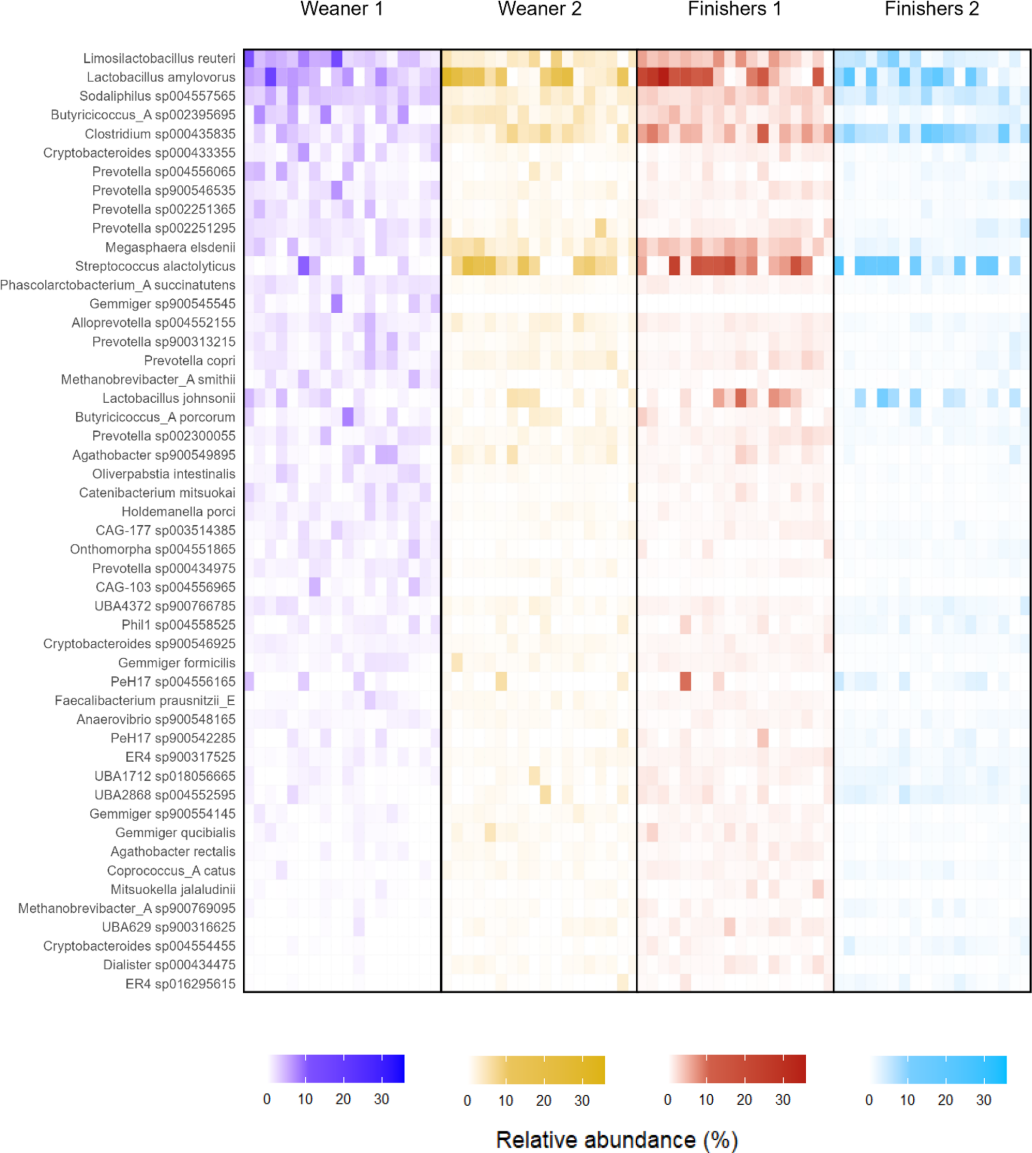
Relative abundance of the core microbiota species per stage. Weaners 1: one week after weaning; Weaners 2: one week prior to transfer to the finisher stage; Finishers 1: one week after transfer to the finisher stage; Finishers 2: one week prior to slaughter. Note: The 50 species in the y axis account for 53% of W1 microbiota composition; 61% of W2; 63% of F1 and 64% of F2

**Fig. 6:**
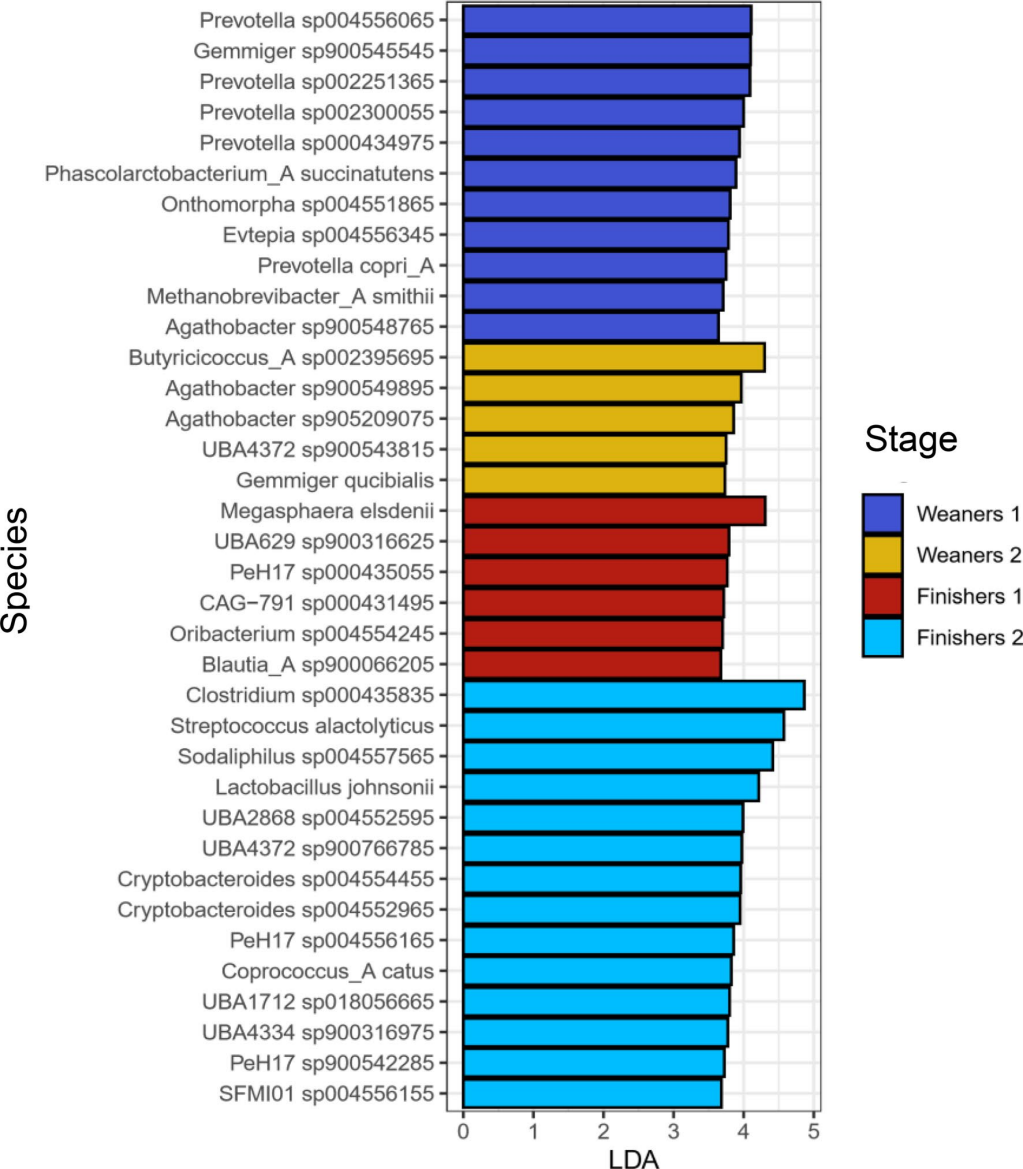
LEfSe (Linear discriminant analysis Effect Size) using stage as class. LDA: linear discriminant analysis; Weaners 1: one week after weaning; Weaners 2: one week prior to transfer to the finisher stage; Finishers 1: one week after transfer to the finisher stage; Finishers 2: one week prior to slaughter

Microbiota composition differed between stages, according to PERMANOVA analysis (Additional file 1), and all pairwise comparisons were significant except between W2 and F1. Ordination plots showed clustering of samples according to age, with large overlapping between W2 and F1 (Fig. 3). Likewise, age was an important clustering factor according to Ward clustering analysis, where four clusters were observed (Fig. 4). Cluster D was essentially composed of samples from W1, while cluster A was dominated by F2. Cluster B was divided into two subclusters, one dominated by F2 and the other dominated by samples from W2 and F1. These latter two stages were closely grouped in cluster C. Contrary to age, farm did not have a marked effect on clustering. With regards to species composition, cluster A included samples from F2 with high proportions of *Streptococcus alactolyticus* and, to a lower extent, of *Lactobacillus johnsonni*, *Clostridium* sp. and *Lactobacillus amylovorus*. Cluster B had high proportions of *Lactobacillus amylovorus*, especially in the subcluster of W2 and F1, while the subcluster with F2 was dominated by *Clostridium* sp. showing negligible proportions of *Streptococcus alactolyticus*. Cluster C was characterized by moderate to high abundances of *Streptococcus alactolyticus*, *Clostridium* sp., *Prevotella hominis* and *Megasphaera elsdenii*. In contrast to clusters A, B and C, cluster D showed higher microbiota evenness and thus species dominance was not as evident. Core microbiota composition at each stage is also shown in Fig. 5. The species explaining differences between stages according to LEfSe are shown in Fig. 6. Using LEfSe, differential abundance analysis between stages identified 11, 5, 6 and 14 species in W1, W2, F1 and F2, respectively.

### Microbiota alpha diversity according to farm characteristics

The univariable modelling results of alpha diversity per stage by farm characteristics are shown in Additional file 2. Most significant associations were observed at stage F1 for species evenness, Shannon diversity and Simpson diversity. Multivariable modelling results of alpha diversity measures using selected farm characteristics as predictors are shown in Table 2. Purchased feed was associated with higher evenness, Shannon and Simpson diversity at W2 (*P* = 0.036), higher richness (*P* = 0.010), Shannon and Simpson diversity (*P* = 0.049) at F2 and higher evenness and Simpson diversity at F1 (*P* = 0.047). Prevalence of *Salmonella* spp. was negatively associated with Simpson diversity at F2 (*P* = 0.035) and tended to be negatively associated with Shannon diversity at F2 (*P* = 0.054) and W2 (*P* = 0.098). Weaner mortality was positively associated with richness at F2 (*P* = 0.016), and Weaner + Finisher mortality showed a positive trend with Shannon diversity at F1 (*P* = 0.085). Age at sale was negatively associated with evenness at W2 (0.050) and AMR tended to be positively associated with Simpson diversity at F1 (*P* = 0.056).

**Table 2:** Multivariable analysis of microbiota alpha-diversity per stage by selected farm characteristics

Outcome	Explanatory variables	Estimate ± SE	*P* value
**Richness F1** Adjusted R^2^: 0.286 *P* = 0.053	Intercept	501.8 ± 69.1	< 0.001*
	Internal Biosecurity	-1.43 ± 0.97	0.161
	Feed form	-28 ± 29	0.352
	ZnOAb	56.5 ± 35.3	0.132
**Richness F2** Adjusted R^2^: 0.483 *P* = 0.006	Intercept	396.4 ± 29.4	< 0.001*
	Weaner mortality	20.6 ± 7.5	0.016*
	Feed origin	-65.8 ± 22.2	0.010*
	Salmonella	-28.2 ± 29.3	0.351
**Evenness W2** Adjusted R^2^: 0.408 *P* = 0.008	Intercept	0.910 ± 0.067	< 0.001*
	Age at sale	-8.2e^− 4^ ± 3.9e^− 4^	0.050*
	Feed origin	-0.025 ± 0.011	0.036*
**Evenness F1** Adjusted R^2^: 0.427 *P* = 0.013	Intercept	0.706 ± 0.019	< 0.001*
	Weaner + Finisher mortality	0.007 ± 0.004	0.111
	AMR	0.026 ± 0.030	0.398
	Feed origin	-0.036 ± 0.016	0.037*
**Shannon W2** Adjusted R^2^: 0.387 *P* = 0.020	Intercept	4.595 ± 0.214	< 0.001*
	Salmonella	-0.197 ± 0.111	0.098
	Internal Biosecurity	0.003 ± 0.004	0.517
	Feed origin	-0.266 ± 0.117	0.040*
**Shannon F1** Adjusted R^2^: 0.419 *P* = 0.024	Intercept	4.390 ± 0.342	< 0.001*
	Weaner + Finisher mortality	0.059 ± 0.032	0.085
	AMR	0.140 ± 0.237	0.563
	Internal Biosecurity	-0.002 ± 0.006	0.683
	Feed origin	-0.258 ± 0.173	0.160
**Shannon F2** Adjusted R^2^: 0.391 *P* = 0.009	Intercept	4.59 ± 0.07	< 0.001*
	Salmonella	-0.27 ± 0.13	0.054*
	Feed origin	-0.21 ± 0.10	< 0.049*
**Simpson W2** Adjusted R^2^: 0.389 *P* = 0.010	Intercept	1.03 ± 0.030	< 0.001*
	Age at sale	0 ± 0	0.133
	Feed origin	-0.01 ± 0.01	0.019*
**Simpson F1** Adjusted R^2^: 0.329 *P* = 0.020	Intercept	0.96 ± 0.01	< 0.001*
	AMR	0.03 ± 0.01	0.056
	Feed origin	-0.02 ± 0.01	0.047*
**Simpson F2** Adjusted R^2^: 0.456 *P* = 0.009	Intercept	0.973 ± 0.014	< 0.001*
	Salmonella	-0.017 ± 0.007	0.035*
	Internal Biosecurity	0 ± 0	0.917
	Feed origin	-0.014 ± 0.008	0.083

Legend: R^2^ - R squared; SE: standard error; Weaners 1: one week after weaning; Weaners 2: one week prior to transfer to the finisher stage; Finishers 1: one week after transfer to the finisher stage; Finishers 2: one week prior to slaughter; AMR: antimicrobial resistance prevalence; ZnOAb: post-weaning use of zinc oxide and medicated feed; Salmonella: prevalence of *Salmonella* spp.; Feed form: liquid or dry; Feed origin: purchased or home-milled

### Microbiota composition according to farm characteristics

**Fig. 7:**
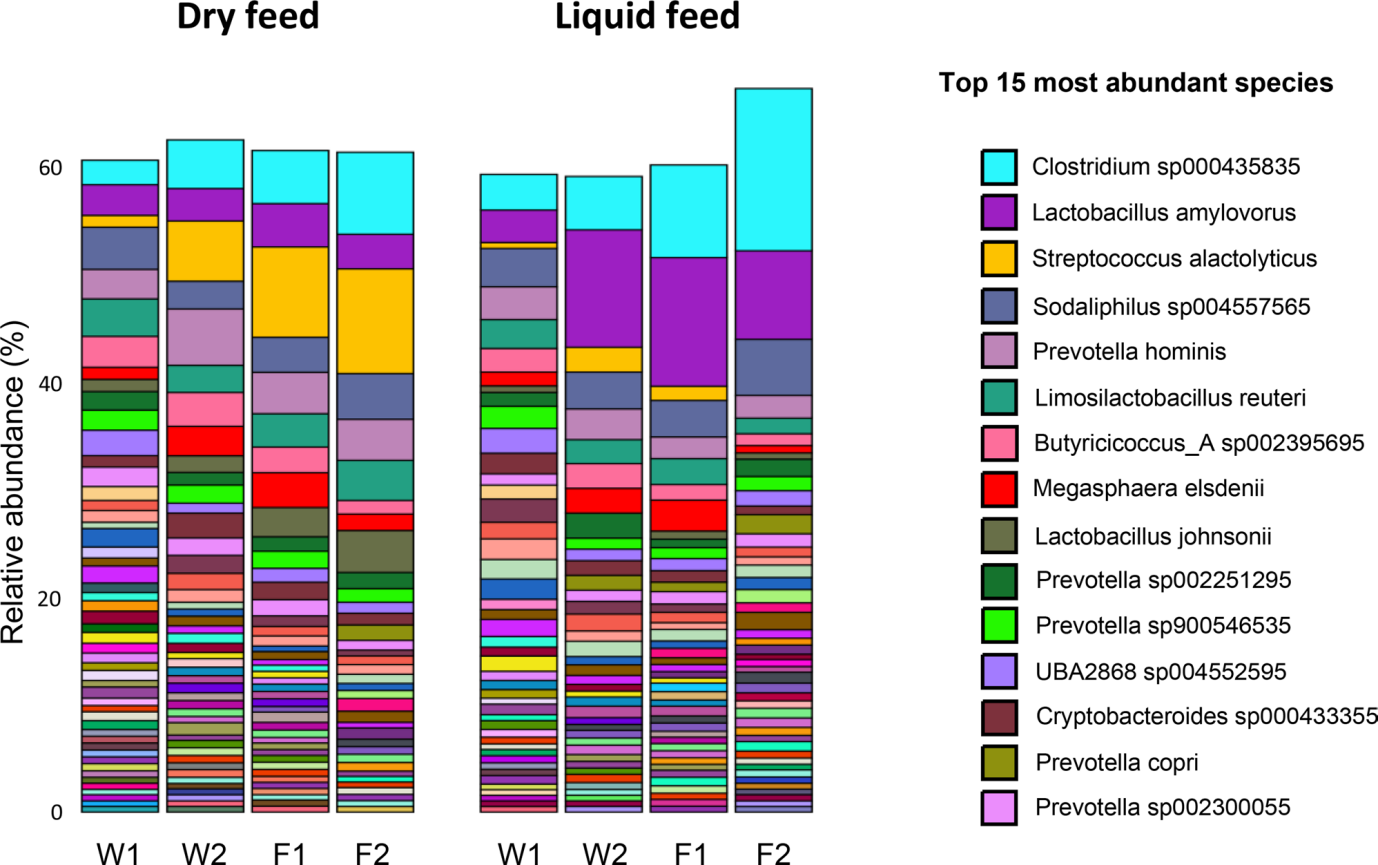
Species relative abundance per stage according to feed form. W1: one week after weaning; W2: one week prior to transfer to the finisher stage; F1: one week after transfer to the finisher stage; F2: one week prior to slaughter. Note: Species with a relative abundance below 5% are not shown

The associations between farm characteristics and microbiota composition at each stage are displayed in Table 3. Microbiota composition at W1 differed according to whether or not farms used zinc oxide and medicated feed (*P* = 0.040). For all other stages, microbiota composition was consistently associated with feed form and feed origin (*P* < 0.050). When modelling microbiota composition of all stages except W1, both stage (R^2^ = 0.18) and feed form (R^2^ = 0.19) were significant predictors (*P* < 0.001). When considering only the two finisher stages, feed form (R^2^ = 0.22, *P* < 0.001) explained more variability than stage (R^2^ = 0.14, *P* = 0.002). Internal biosecurity was a predictor at F1 and F2 (*P* < 0.050), while external biosecurity was a predictor at F2 (*P* = 0.045) and showed a tendency at F1 (*P* = 0.055). Piglet mortality was a predictor at F1 (R^2^ = 0.15, *P* = 0.033) and F2 (R^2^ = 0.19, *P* = 0.005). Core microbiota composition was strongly associated with feed form (Fig. 7). For animals on a dry feed diet, *Streptococcus alactolyticus* was the most predominant species and its relative abundance increased according to age, from 5.2% at W2 to 9.3% at F2. In contrast, liquid fed pigs had low abundances of this species, which decreased with age, from 2.6% at W2 to under 0.5% at F2. These animals showed a microbiota composition where *Lactobacillus amylovorus* and *Clostridium* sp. were predominant, the latter increasing from 5% at W2 to 14.7% at F2. Both species were also among the most predominant for dry fed pigs, representing, however, smaller proportions of the microbiota.

**Table 3:** PERMANOVA analysis of microbiota composition according to farm characteristics for each stage, reported as P value (R^2^)

	microbiota W1	microbiota W2	microbiota F1	microbiota F2
ZnOAb	**0.040 (0.14)**	0.990 (0.02)	0.409 (0.06)	0.769 (0.03)
PRRS	0.610 (0.04)	0.541 (0.05)	0.273 (0.07)	0.587 (0.04)
Piglet mortality	0.225 (0.08)	0.091 (0.11)	**0.033 (0.15)**	**0.005 (0.19)**
Weaner + Finisher mortality	0.557 (0.05)	0.561 (0.04)	0.13 (0.09)	0.156 (0.09)
Weaner mortality	0.484 (0.05)	0.792 (0.03)	0.210 (0.08)	0.086 (0.11)
Finisher mortality	0.734 (0.04)	0.438 (0.05)	0.200 (0.08)	0.504 (0.05)
Pigs/sow/year	0.485 (0.05)	0.572 (0.05)	0.066 (0.12)	0.216 (0.08)
Age at sale	0.215 (0.08)	0.822 (0.03)	0.513 (0.05)	0.890 (0.02)
Daily gain	0.211 (0.08)	0.844 (0.03)	0.238 (0.08)	0.569 (0.04)
Salmonella	0.951 (0.02)	0.316 (0.07)	0.135 (0.10)	0.142 (0.10)
AMR	0.605 (0.04)	0.453 (0.05)	0.136 (0.10)	0.628 (0.04)
External Biosecurity	0.476 (0.05)	0.256 (0.07)	0.055 (0.13)	**0.045 (0.13)**
Internal Biosecurity	0.200 (0.08)	0.159 (0.09)	**0.006 (0.20)**	**0.017 (0.17)**
Feed form	0.557 (0.04)	**0.007 (0.18)**	**0.001 (0.27)**	**< 0.001 (0.24)**
Feed origin	0.222 (0.07)	**0.027 (0.14)**	**< 0.001 (0.30)**	**< 0.001(0.29)**

Legend: W1: one week after weaning; W2: one week prior to transfer to the finisher stage; F1: one week after transfer to the finisher stage; F2: one week prior to slaughter; AMR: antimicrobial resistance; PRRS: porcine reproductive and respiratory syndrome; Salmonella: prevalence of *Salmonella* spp.; ZnOAb: post-weaning use of zinc oxide and medicated feed

### Microbiome functional profiles

**Fig. 8:**
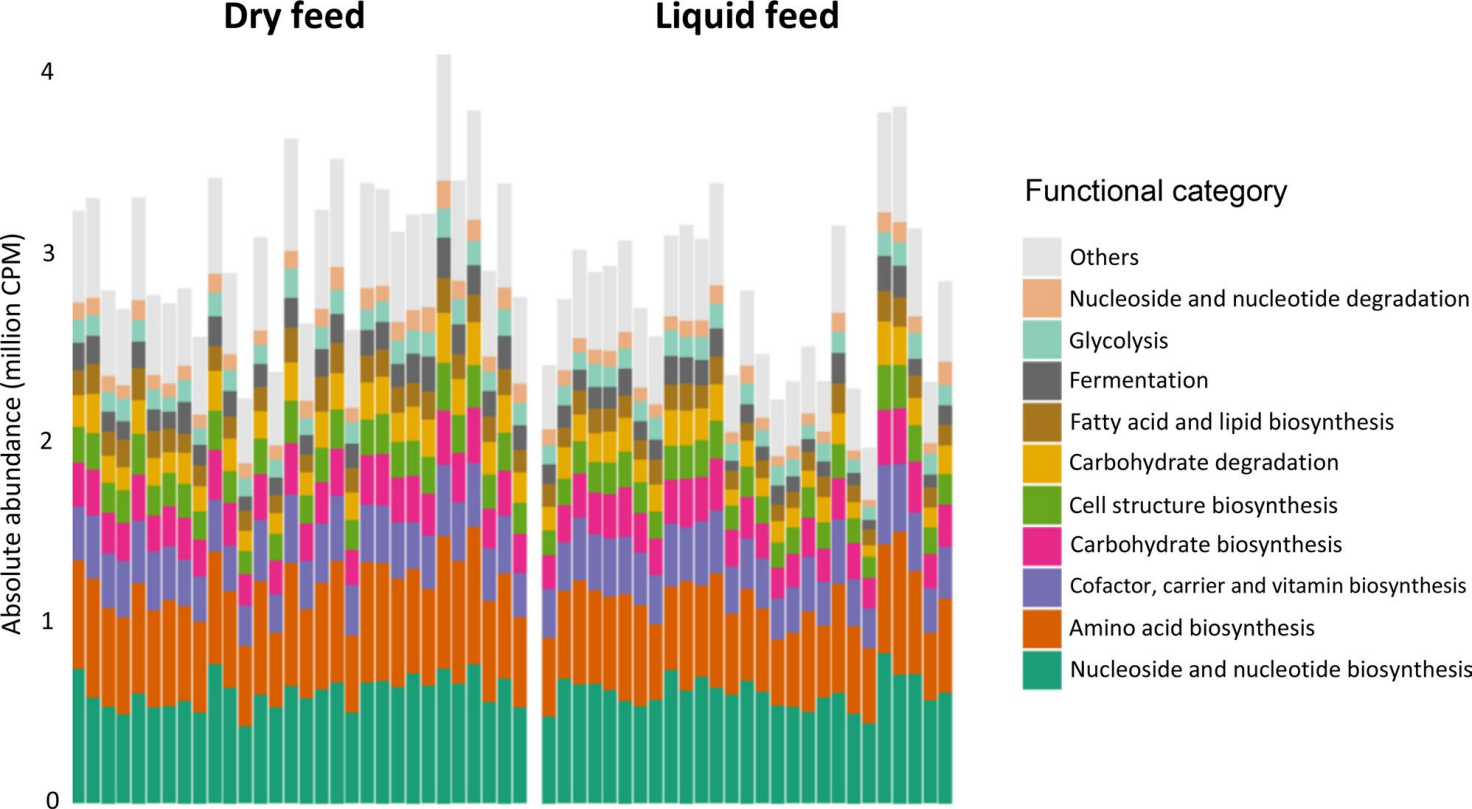
Relative abundance of microbiome functional profiles at individual level by feed form. Data were aggregated by functional categories using Superclass 2 from the microeco package. Samples from stage W1 (one week after weaning) were omitted as animals at this stage were fed a dry diet. CPM: counts per million

**Fig. 9:**
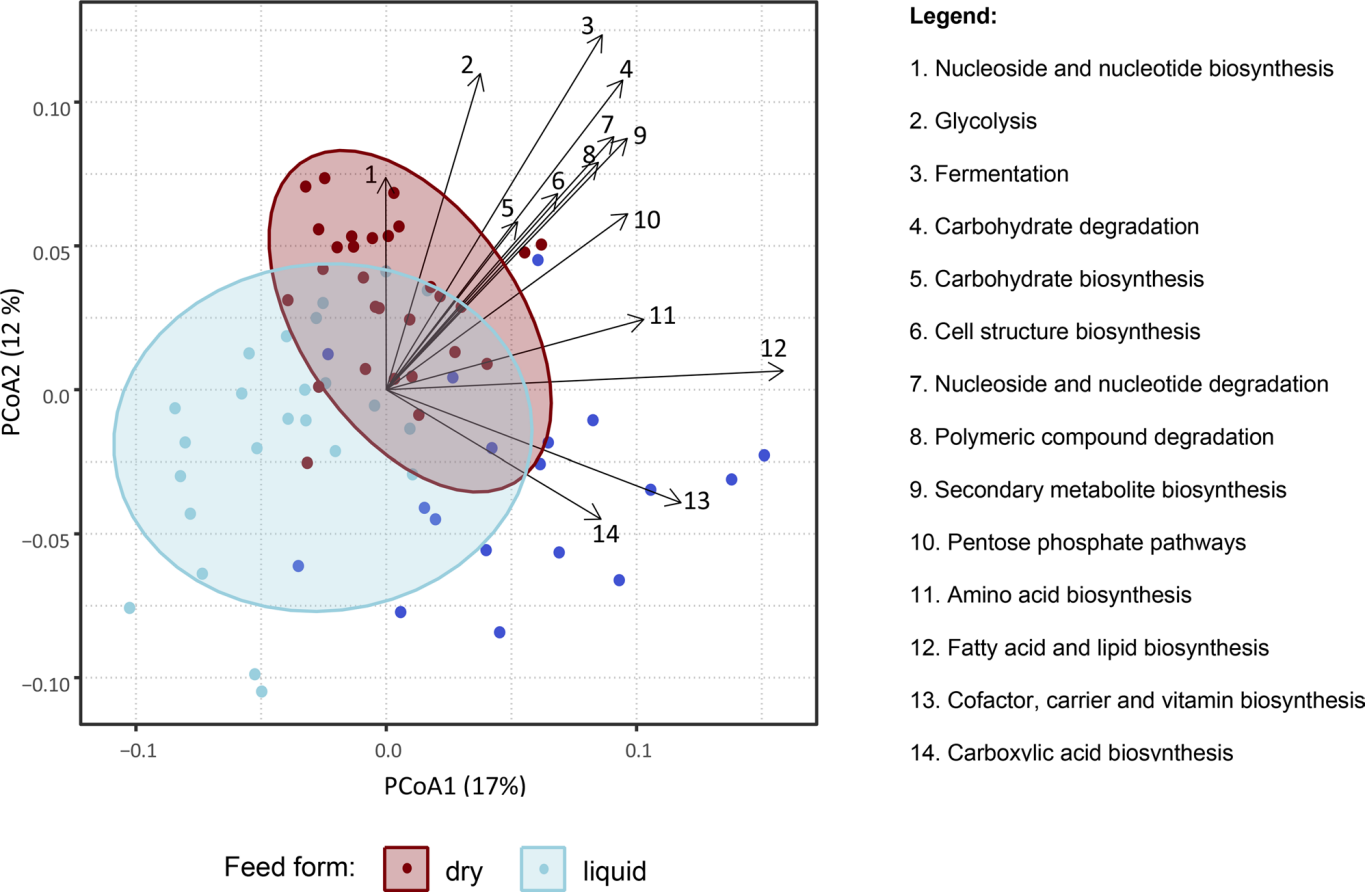
PCoA representation of microbiome functional categories according to feed form based on Bray-Curstis distances of Hellinger transformed reads (counts per million) between samples. Samples in dark blue indicate the stage W1 (one week after weaning), where all farms used a dry diet. The main functional pathways influencing the ordination were analysed using the envfit function from the vegan package. Data were aggregated by functional categories using Superclass 2 from the microeco package

Functional gene analysis identified 510 genes which were grouped into ten main functional super classes, of which the most abundant were nucleoside and nucleotide biosynthesis, amino acid biosynthesis, and cofactor, carrier and vitamin biosynthesis (Fig. 8). Differences according to stage were observed according to PERMANOVA analysis (Additional file 5) and PCoA representations (Additional file 6). All pairwise differences between W1 and other stages were significant, and this stage was associated with higher abundances of “cofactor, carrier and vitamin biosynthesis” and “carboxylic acid biosynthesis” categories (Fig. 9). Feed form was also associated with functional categories, with samples from animals on dry feed showing higher abundances of functional profiles such as glycolysis, fermentation and carbohydrate degradation (Fig. 9).

### Resistome

**Fig. 10:**
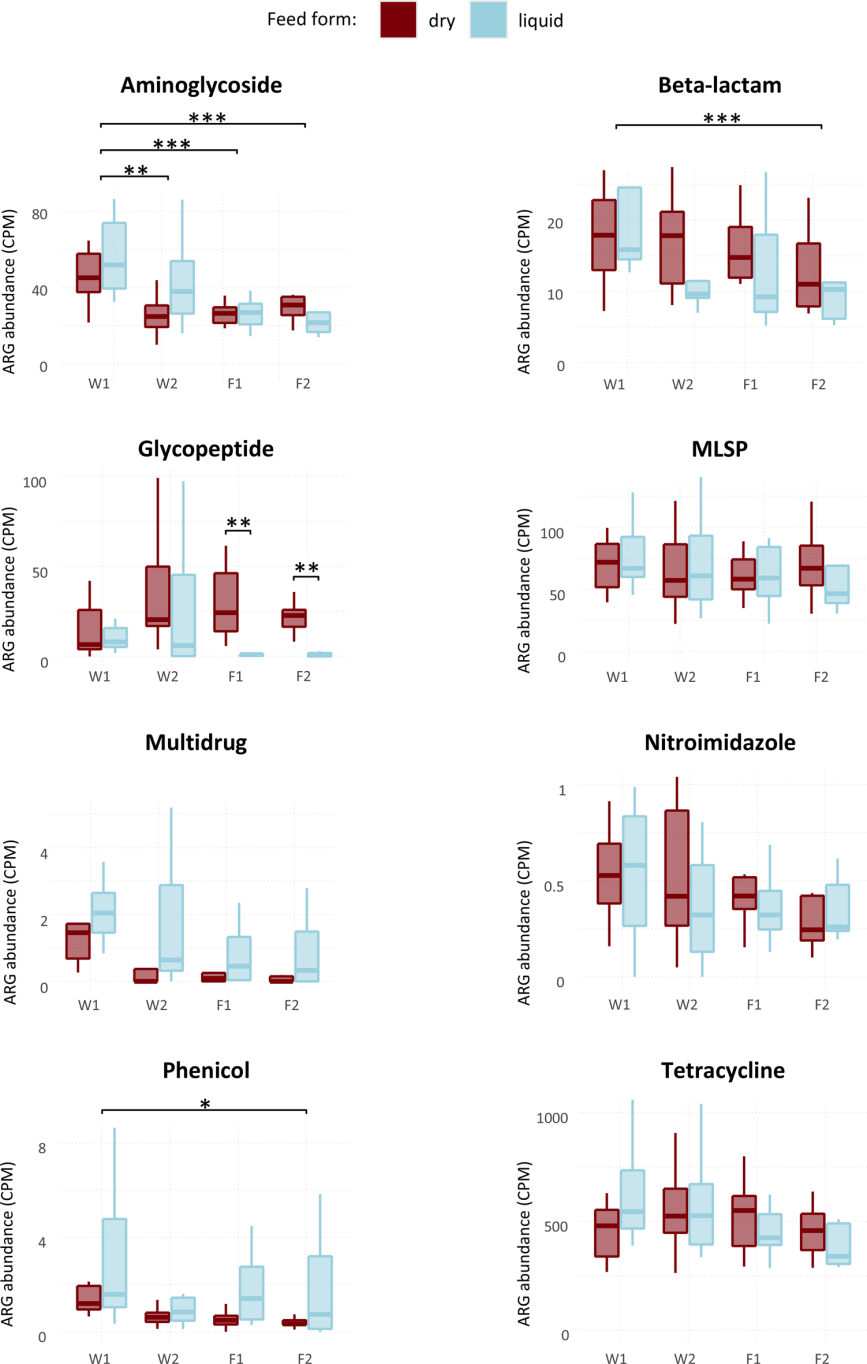
Abundance of antimicrobial resistance genes (counts per million) aggregated by antimicrobial class, according to stage and feed form. MLSP: macrolide, lincosamide, streptogramin and pleuromutilin; W1: one week after weaning; W2: one week prior to transfer to the finisher stage; F1: one week after transfer to the finisher stage; F2: one week prior to slaughter; *: *P* < 0.05; **: *P* < 0.01

A total of 282 ARGs were identified, belonging to 122 gene families conferring resistance to 12 antimicrobial classes. When considering all samples, the resistome was strongly dominated by tetracycline resistance genes (37,969 CPM), followed by MLSP (macrolide-lincosamide-streptogramin-pleuromutilin) (4,980 CPM) and aminoglycosides (2,849 CPM). Differences of ARG abundance between stages were detected for aminoglycoside, beta-lactam and phenicol classes, with W1 showing higher abundances than other stages (Fig. 10). According to PERMANOVA analysis, all pairwise differences observed were between W1 and the other three stages (Additional file 3). Ordination plots also showed clustering of samples according to stage and feed form (Additional file 4). Feed form was also associated with ARG abundance, as glycopeptide resistance was higher in F1 and F2 on dry feed in comparison with liquid feed (Fig. 10). In terms of alpha diversity, ARG richness was positively associated with mortality at all stages and with AMR prevalence and negatively associated with prevalence of *Salmonella* spp.

## Discussion

The gut microbiome is regarded as an important source of information to better understand and improve health and growth in animals, often studied through faecal microbiota. In pigs, most microbiome studies have taken place in research facilities, which are characterized by a high health status and operate under strictly controlled conditions [8, 19]. While such studies achieve a good control of background variability and are necessary to advance knowledge, it is also relevant to consider the effects of management and health on the microbiome in commercial farms. This would enhance the application of knowledge generated in research facilities in commercial pig farms. The current cross-sectional study characterized the faecal microbiota of pigs at selected production stages across eighteen farms. On each farm, pigs were sampled at four stages, in order to study the temporal dynamics of the microbiome across the production cycle, with particular emphasis on the effects of weaning and relocation. Microbiota composition, alpha and beta diversities were described and their associations with health status, productive performance and farm characteristics were investigated. It was hypothesized that the contrasting differences between farms would be reflected in the microbiota of their animals.

Although temporal dynamics of the microbiome are better characterized through longitudinal studies, the current cross-sectional approach can inform on some microbiota changes across the production cycle. Age was an important factor to explain variation in microbiota alpha-diversity measured in terms of species richness, evenness and Simpson diversity, whereas no differences were noted in Shannon diversity. A consistent observation across all alpha diversity indices was a large within-stage variability (Fig. 1). While this was an expected consequence of studying multiple farms with contrasting characteristics, within-group variability is a common observation in studies taking place on a single farm [47–50]. It was still possible, however, to detect pairwise differences between stages, where richness increased with age while evenness and Simpson diversity decreased. The increasing number of species across the production cycle is in line with several studies, especially when comparing piglets in the lactation stage, or recently weaned, with older pigs [47, 51, 52]. In contrast, Massacci et al. observed no differences in richness when comparing the microbiota one week after weaning and at sixty days of age, for pigs weaned at two, three, four and six weeks of age [53].

In terms of diversity, Shannon and Simpson indices showed different results, which is not uncommon in microbiome studies, due to the different sensitivity of each index for richness and evenness [54, 55]. The increase in richness but lack thereof in Shannon diversity is in line with the findings of Gaire at al., who did not observe consistent differences between weaning and thirteen weeks of age [52]. Likewise, Massacci et al. reported no differences in Shannon diversity between one week after weaning and sixty days of age, across pigs weaned at four different ages [53]. On the other hand, Shannon diversity has also been reported to increase throughout the production cycle [51]. Our study suggests that throughout the production cycle, the pig’s microbiota becomes richer and less even, the latter resulting from an increase in the dominance of certain species with age (Fig. 2).

According to PERMANOVA, microbiota composition was strongly associated with stage (*P* < 0.001, R^2^ = 0.34). This was expected since age is one of the most common factors explaining variation in microbiome datasets [8]. All pairwise differences between stages were significant except between W2 and F1. A possible contributing factor was the close proximity of two weeks between the two stages, whereas any other two stages were set apart by at least five weeks. Despite this, microbiota composition can change over short time periods, especially in the presence of environmental factors like relocation and diet changes [8, 19], both of which take place when animals are transferred to the finisher stage. The similarities in microbiota alpha and beta diversity between W2 and F1 may therefore suggest that at the end of the weaner stage (W2) the microbiota has reached a certain level of stability that confers resilience against environmental stressors. Similar results were obtained by Ward clustering analysis, where differential sample composition caused W1 to cluster together and to be clearly separated from other stages (Fig. 4). Samples from F2 also clustered together, though to a lesser extent. With regards to differentially abundant species per stage (Fig. 6), these should be interpreted with caution as LEfSe is prone to false positive results. Unlike the transfer to the finisher stage, weaning is characterized by mixing immunologically vulnerable animals and moving them into new facilities, with significant changes in diet and exposure to novel environmental stressors [56]. The resulting changes in the microbiome of the early weaned pig often include gut dysbiosis and diarrhoea [10, 57]. While the effects of weaning on the microbiota would have been better captured by including a pre-weaning sampling stage, this was not carried out due to practical and welfare constraints associated with obtaining a relevant number of faecal samples at lactation. Nevertheless, with the exception of Shannon diversity, the most contrasting differences between stages, for both alpha and beta diversity, were observed between W1 and other stages. Further, the high Simpson diversity at W1 is likely a result from an even and unstable microbiota undergoing adaptation, and should not be interpreted under the frequent assumption that diversity and health are positively related. Indeed, associations between microbiota diversity and health require careful interpretation [54, 55, 58]. As such, this study aligns with the general theory that post-weaning changes in the porcine gut microbiota are more evident in younger stages of growth, and generally involve an increasing trend of alpha diversity and a tendency towards compositional stability over time [19].

As stated above, an expected outcome of this study was to observe differences in microbiota according to farm characteristics. Microbiota richness and diversity at F2 were negatively associated with prevalence of *Salmonella* spp. While the associations between prevalence of *Salmonella* spp. and microbiota are not clear [20, 59], reduced diversity can be a risk factor for gut colonization by opportunistic microbes [60, 61]. In the present study, the decrease in diversity with age may have favoured gut colonization by *Salmonella* spp. On the other hand, it cannot be excluded that the lower richness and diversity at F2 were a result of, rather than a cause for, higher prevalence of *Salmonella* spp. Nonetheless, this association requires careful interpretation as there are several complexities concerning infection by *Salmonella* spp. that were not taken into account. While the influence of diet on the porcine gut microbiota has been studied, there is a lack of knowledge regarding how diet induced changes in the microbiota are associated with prevalence of *Salmonella* spp. and other gastrointestinal pathogens.

In this study, prevalence of *Salmonella* spp. was higher in farms doing home-milling (*P* = 0.01) and tended to be higher in farms using liquid feed (*P* = 0.07). With regards to the latter, it is worth mentioning that although liquid feed has been associated with lower prevalence of *Salmonella* spp., due to its lower pH, fermentation of liquid feed is not a common practice in Irish farms, unlike what happens in other countries such as in Northern Europe. It should also be noted that all farms using liquid feed milled their own feed, whereas dry feed was mostly purchased. Although it is difficult to determine whether feed form or feed origin were more influential for prevalence of *Salmonella* spp., the latter was more important according to multivariable analysis (Table 2). Contrary to home-milled feed, purchased feed is usually obtained from suppliers that regularly test feed for presence of *Salmonella* spp. Thus, non-fermented home-milled liquid feeding systems may contribute to the persistence of *Salmonella* spp. in pig farms. The results of this study highlight the importance of further investigating the associations between feeding management, gut microbiota and gastrointestinal pathogens. This assumes particular relevance from a public health standpoint, as salmonellosis is among the main foodborne diseases worldwide, and often associated with consumption of pig meat [62]. Manipulation of diet has long been known as a control measure to reduce shedding of *Salmonella* spp. in pigs [63] but further insight into the mechanisms behind this would potentially facilitate more effective and less costly use of this intervention. It is also important to take into account other factors such as gastrointestinal disease incidence and vaccination.

Diversity at all stages after W1, as well as richness at F2, were higher in farms purchasing feed, in comparison with those home-milling it. Due to the importance of feed as a driver of the microbiome, our farm selection included eight farms which purchased feed and ten home-milling feed. A notable difference between the two sources lies in the higher complexity of purchased feed, as it includes more ingredients. Dietary diversity (or variety) is considered a key contributing factor for a desirable microbiome with higher alpha diversity [64, 65]. It is therefore possible that, in the present study, the more complex (purchased) feed promoted the development of a more diverse microbiota. The effects of feed form (dry vs. liquid) were more evident in terms of microbiota composition as described below.

Post weaning use of zinc oxide and medicated feed was the only factor associated with microbiota composition at W1 (Table 3). While the number of farms not using ZnOAb was low (three), the differential microbiota composition was expected, as both zinc oxide and in-feed antimicrobials are known to shape the microbiota of weaned pigs [13, 66]. While the absence of associations between ZnOAb and alpha-diversity may have been affected by the low number of farms, studies have suggested that in-feed treatments impact microbiota composition to a greater extent when compared to alpha-diversity [13, 66]. In spite of the contrasting mortality and growth figures between some farms in our study, these were not reflected in terms of microbiota composition according to PERMANOVA analysis. Although some differences were observed according to internal biosecurity, piglet mortality and especially both feed type and feed origin, it is possible that the variability introduced by sampling multiple farms affected the determination of differences according to growth and mortality. With regards to biosecurity and piglet mortality, differences were observed at all stages except W1. Both variables are strongly associated with management practices in farms, highlighting the influence of husbandry practices on the gut microbiota. The absence of differences at W1 may have resulted from the marked disruption of the gut microbiota at weaning in all farms. The associations between microbiota composition and both feed form and feed origin were expected, given the role of feed in shaping the gut microbiome and the different microbial composition between dry and liquid feed, the latter with higher concentration of lactic acid bacteria [67]. Interestingly, feed form (liquid or dry) explained more variability than age, in terms of microbiota composition of all stages except W1. The influence of feed on shaping the gut microbiome was further highlighted by the differences in the functional profiles (Figs. 8 and 9) and resistome (Fig. 10) between dry fed and liquid fed pigs. Further research is needed to clarify how nutrition and microbiota influence the occurrence of ARGs. The higher abundance of functional profiles such as glycolysis, fermentation and carbohydrate degradation in dry fed pigs could result from the higher nutrient density of solid diets in comparison with liquid diets and resulting differences in digestibility. There is a lack of studies comparing the effects of liquid and dry feed on the gut microbiota using high throughput sequencing data as opposed to culture-based microbiology techniques [67]. He, et al. reported lower abundances of *Streptococcus* and *Clostridium* but higher abundances of *Lactobacillus*, at genus level, in weaned piglets fed a fermented liquid diet, compared to those on a pelleted diet, using digesta samples [68]. Although these animals were 32 days old and further results were not reported, a similar pattern was observed in our study from stage W2 onwards, except for *Clostridium* which was more abundant in liquid fed finisher pigs.

Given the cross-sectional design of the study, it is important to interpret the results related to microbiome temporal dynamics with caution, as different batches of animals were sampled at each farm. Nevertheless, the findings of this study are an important contribution to determine the factors that shape the faecal microbiota of pigs on commercial farms. Further, the associations between feeding management and microbiome can inform on farm management decisions regarding risk mitigation of *Salmonella* spp. The consistent patterns observed across farms with contrasting characteristics, suggest that core faecal microbiome in pigs on commercial farms is primarily driven by diet and age, rather than farm indicators of health, as measured by mortality and growth, and antimicrobial resistance.

## Conclusions

In this cross-sectional study age and diet were the main drivers of microbiota, resistome and functional profiles on commercial pig farms. The fact that the most evident differences were observed between weaned pigs and older animals suggests that microbiota is most affected by weaning, thereafter evolving towards stability, which is in line with previous studies. Purchased feed was associated with a richer and more diverse microbiota, probably for having more ingredients than home-milled feed, while feed form (liquid or dry) explained more variability in microbiota composition than age, especially for finishers. Associations between microbiome, prevalence of *Salmonella* spp. and diet suggest that diet induced changes in the microbiota may be a determining factor in gut colonization and infection by pathogens.

## Electronic Supplementary Material

Below is the link to the electronic supplementary material.

Additional file 1. PERMANOVA analysis of microbiota composition according to stage.

Additional file 2. Univariable analysis of microbiota alpha-diversity per stage by farm characteristics (P values).

Additional file 3. PERMANOVA analysis of resistome (antimicrobial resistance gene abundance) according to stage.

Additional file 4. PCoA representation of the resistome according to stage (A) and feed form (B), based on Bray-Curtis distances of Hellinger transformed normalized read counts (counts per million).

Additional file 5. PERMANOVA analysis of microbiome functional profiles according to stage.

Additional file 6. PCoA representation of microbiome functional profiles according to stage, based on Bray-Curtis distances of Hellinger transformed normalized read counts (counts per million).

## Data Availability

Sequence data from all samples, including negative and positive controls and the associated sample metadata have been submitted to NCBI Sequence Read Archive (SRA) and will be available under Bioproject accession PRJNA1211805 at: https://dataview.ncbi.nlm.nih.gov/object/PRJNA1211805?reviewer=k5hj9obuu96rd4i1j0eb7855ki.

## Abbreviations

AMR: Antimicrobial resistance
ESBL: Extended-spectrum beta-lactamases
CPM: counts per million
F1: One week after transfer to the finishing stage
F2: One week prior to slaughter
LEfSe: Linear discriminant analysis effect size
MLSP: macrolide, lincosamide, streptogramin and pleuromutilin
MSRV: Modified semi-solid rappaport-vassiliadis
PCoA: Principal coordinate analysis
PERMANOVA: Permutational multivariate analysis of variance
PRRS: Porcine respiratory and reproductive syndrome
SE: Standard error
TBX: Tryptone bile X-glucuronide
W1: One week after weaning
W2: One week prior to transfer to the finishing stage
XLD: Xylose lysine deoxycholate
ZnO: Zinc oxide
ZnOAb: Post-weaning use of zinc oxide and in-feed antimicrobials
