# Supplementary material for: Cross-sectional study characterizing the porcine faecal microbiome in commercial farms
Source: Porcine Health Manag. 2026 Jan 22;12:1. doi: 10.1186/s40813-025-00480-3 (PMC12828960; doi:10.1186/s40813-025-00480-3)
Supplement: Supplementary file 2 — Additional file 2. Univariable analysis of microbiota alpha-diversity per stage by farm characteristics (P values). [file 40813_2025_480_MOESM2_ESM.docx]

**Additional file 2. Univariable analysis of microbiota alpha-diversity per stage by farm characteristics (P values).**

|  | **Species Richness** | | | | **Species Evenness** | | | | **Shannon Diversity** | | | | **Simpson Diversity** | | | |
| --- | --- | --- | --- | --- | --- | --- | --- | --- | --- | --- | --- | --- | --- | --- | --- | --- |
| **Factor** | **W1** | **W2** | **F1** | **F2** | **W1** | **W2** | **F1** | **F2** | **W1** | **W2** | **F1** | **F2** | **W1** | **W2** | **F1** | **F2** |
| **ZnOAb** | 0.30 | 0.91 | 0.06 | 0.29 | 0.84 | 0.17 | 0.07 | 0.69 | 0.38 | 0.22 | **0.03** | 0.47 | 0.49 | 0.30 | **0.02** | 0.83 |
| **PRRS** | 0.47 | 0.90 | 0.67 | 0.75 | 0.43 | 0.64 | 0.10 | 0.31 | 0.39 | 0.71 | 0.26 | 0.60 | 0.39 | 0.58 | 0.25 | 0.58 |
| **Piglet mort.** | 0.91 | 0.40 | 0.56 | 0.12 | 0.98 | 0.10 | **<0.01** | 0.33 | 0.86 | 0.08 | **0.02** | 0.20 | 0.77 | 0.11 | **0.01** | 0.33 |
| **W+F mort.** | 0.71 | 0.19 | 0.22 | 0.12 | 0.56 | 0.65 | **0.04** | 0.23 | 1 | 0.32 | **0.04** | 0.15 | 0.67 | 0.52 | 0.14 | 0.20 |
| **Weaner mort.** | 0.95 | 0.36 | 0.36 | 0.09 | 0.78 | 0.91 | **0.04** | 0.25 | 0.91 | 0.61 | 0.06 | 0.15 | 0.65 | 0.91 | 0.11 | 0.25 |
| **Finisher mort.** | 0.43 | 0.13 | 0.17 | 0.27 | 0.42 | 0.44 | 0.08 | 0.29 | 0.91 | 0.17 | 0.06 | 0.26 | 0.75 | 0.25 | 0.28 | 0.23 |
| **Pigs/sow/year** | **0.03** | **<0.01** | 0.99 | 0.10 | 0.29 | 0.44 | 0.66 | 0.37 | **0.05** | 0.55 | 0.75 | 0.20 | 0.27 | 0.48 | 0.90 | 0.31 |
| **Age at sale** | 0.60 | 0.46 | 0.70 | 0.53 | 0.20 | **0.02** | 0.49 | 0.99 | 0.35 | 0.16 | 0.51 | 0.79 | 0.45 | **0.05** | 0.58 | 0.78 |
| **Daily gain** | 0.39 | 0.21 | 0.67 | 0.17 | 0.54 | 0.18 | 0.13 | 0.37 | 0.42 | 0.65 | 0.19 | 0.24 | 0.67 | 0.31 | 0.22 | 0.32 |
| **Salmonella** | 0.48 | **0.04** | 0.53 | 0.08 | 0.74 | 0.22 | 0.10 | **0.02** | 0.89 | **0.04** | 0.14 | **0.02** | 0.60 | 0.20 | 0.20 | **0.01** |
| **AMR** | 0.84 | 0.75 | 0.27 | 0.66 | 0.64 | 0.69 | **0.01** | 0.66 | 0.79 | 0.69 | **0.02** | 0.67 | 0.91 | 0.37 | **0.05** | 0.53 |
| **External BC** | 0.51 | 0.63 | 0.13 | 0.14 | 0.81 | 0.74 | **<0.01** | 0.17 | 0.76 | 0.66 | **<0.01** | 0.13 | 0.70 | 0.80 | **0.01** | 0.15 |
| **Internal BC** | 0.99 | 0.12 | **0.04** | 0.23 | 0.25 | 0.27 | 0.19 | 0.06 | 0.50 | 0.10 | 0.08 | 0.07 | 0.26 | 0.18 | 0.16 | **0.04** |
| **Feed form** | 0.47 | 0.66 | 0.07 | 0.42 | 0.29 | 0.19 | 0.15 | 0.81 | 0.30 | 0.22 | 0.08 | 0.62 | 0.49 | 0.06 | **0.04** | 0.33 |
| **Feed origin** | 0.99 | 0.20 | 0.13 | **0.03** | 0.14 | **0.01** | 0.09 | **0.04** | 0.40 | **<0.01** | 0.07 | **0.02** | 0.22 | **<0.01** | **0.04** | **<0.01** |

Weaners 1: one week after weaning; Weaners 2: one week prior to transfer to the finisher stage; Finishers 1: one week after transfer to the finisher stage; Finishers 2: one week prior to slaughter; AMR: antimicrobial resistance; BC: Biosecurity; mort: mortality; PRRS: porcine reproductive and respiratory syndrome; Salmonella: prevalence of *Salmonella* spp.; W+F: sum of the mortality in the weaner and finisher stages; ZnOAb: post-weaning use of zinc oxide and medicated feed.
