# Supplementary material for: Cross-sectional study characterizing the porcine faecal microbiome in commercial farms
Source: Porcine Health Manag. 2026 Jan 22;12:1. doi: 10.1186/s40813-025-00480-3 (PMC12828960; doi:10.1186/s40813-025-00480-3)
Supplement: Supplementary file 3 — Additional file 3. PERMANOVA analysis of resistome (antimicrobial resistance gene abundance) according to stage. [file 40813_2025_480_MOESM3_ESM.docx]

**Additional file 3**. **PERMANOVA analysis of resistome (antimicrobial resistance gene abundance) according to stage.**

|  | Adjusted P-value | R^2^ |
| --- | --- | --- |
| Stage | <0.001 | 0.15 |
| W1 vs W2 | 0.018 | 0.12 |
| W1 vs F1 | 0.018 | 0.19 |
| W1 vs F2 | 0.018 | 0.17 |
| W2 vs F1 | 1.000 | 0.02 |
| W2 vs F2 | 1.000 | 0.03 |
| F1 vs F2 | 1.000 | 0.02 |

Weaners 1: one week after weaning; Weaners 2: one week prior to transfer to the finisher stage; Finishers 1: one week after transfer to the finisher stage; Finishers 2: one week prior to slaughter
