# Supplementary material for: Cross-sectional study characterizing the porcine faecal microbiome in commercial farms
Source: Porcine Health Manag. 2026 Jan 22;12:1. doi: 10.1186/s40813-025-00480-3 (PMC12828960; doi:10.1186/s40813-025-00480-3)
Supplement: Supplementary file 4 — Additional file 4. PCoA representation of the resistome according to stage (A) and feed form (B), based on Bray-Curtis distances of Hellinger transformed normalized read counts (counts per million). [file 40813_2025_480_MOESM4_ESM.docx]

**Additional file 4. PCoA representation of the resistome according to stage (A) and feed form (B), based on Bray-Curtis distances of Hellinger transformed normalized read counts (counts per million).**

Glycopeptide

Sulfonamides

Trimethoprim

Aminoglycoside

Tetracycline

−0.1

0.0

0.1

0.2

−0.2

−0.1

0.0

0.1

0.2

PCoA1 (22%)

PCoA2 (9 %)

Beta-lactam

Feed form:

dry

liquid

Stage:

Weaners 1

Weaners 2

Finishers 1

Finishers 2

−0.1

0.0

0.1

0.2

−0.2

−0.1

0.0

0.1

0.2

PCoA2 (9 %)

PCoA1 (22%)

**A)**

**B)**

Weaners 1: one week after weaning; Weaners 2: one week prior to transfer to the finisher stage; Finishers 1: one week after transfer to the finisher stage; Finishers 2: one week prior to slaughter.
