# Supplementary material for: Cross-sectional study characterizing the porcine faecal microbiome in commercial farms
Source: Porcine Health Manag. 2026 Jan 22;12:1. doi: 10.1186/s40813-025-00480-3 (PMC12828960; doi:10.1186/s40813-025-00480-3)
Supplement: Supplementary file 6 — Additional file 6. PCoA representation of microbiome functional profiles according to stage, based on Bray-Curtis distances of Hellinger transformed normalized read counts (counts per million). [file 40813_2025_480_MOESM6_ESM.docx]

**Additional file 6. PCoA representation of microbiome functional profiles according to stage, based on Bray-Curtis distances of Hellinger transformed normalized read counts (counts per million).**

Stage:

Weaners 1

Weaners 2

Finishers 1

Finishers 2

−0.10

−0.05

0.00

0.05

0.10

−0.10

−0.05

0.00

0.05

0.10

0.15

PCoA2 (12 %)

PCoA1 (17 %)

Weaners 1: one week after weaning; Weaners 2: one week prior to transfer to the finisher stage; Finishers 1: one week after transfer to the finisher stage; Finishers 2: one week prior to slaughter.
